# Risk Factors of Growth Retardation and Developmental Deficits in Very Preterm Infants in a German Tertiary Neonatal Unit

**DOI:** 10.3390/children8050394

**Published:** 2021-05-14

**Authors:** Hanne Lademann, Anna Janning, Josephyn Müller, Luisa Neumann, Dirk Olbertz, Jan Däbritz

**Affiliations:** 1 Department of Pediatrics, Rostock University Medical Center, 18057 Rostock, Germany; jan.daebritz@med.uni-rostock.de; 2 Medical School, Rostock University Medical Center, 18055 Rostock, Germany; anna.janning@uni-rostock.de (A.J.); luisa.neumann91@gmail.com (L.N.); 3 Department of Neonatology, Böblingen Children’s Hospital Perinatal Centre Level 3, Klinikverbund Südwest, 71032 Böblingen, Germany; josephyn.mueller@googlemail.com; 4 Department of Neonatology, Südstadt Hospital Rostock, 18059 Rostock, Germany; dirk.olbertz@kliniksued-rostock.de

**Keywords:** catch-up growth, preterm infants, psychomotor development, neurodevelopment, outcome, Bayley scales of infant development, BSID, developmental delay

## Abstract

Over the last two decades, improvements in perinatology have led to increased survival rates of preterm infants. A large number of studies and meta-analyses have investigated of preterm infants and/or the influence of developmental care. However, the combined influence of the most frequent risk factors and developmental care on the long-term somatic, motor, and cognitive outcome of preterm infants remains unclear. This retrospective, single-center cohort study includes 256 children treated in a tertiary neonatal intensive care unit in Rostock, Germany, between 2008 and 2013. Follow-up examinations (somatic, psychomotor, and mental development) were performed at (corrected) 24 months using Bayley Scales of Infant Development II (BSID-II). Developmental care was carried out according to the legal framework and national guidelines (physiotherapy and/or early education). Bronchopulmonary dysplasia (BPD) and an exclusive formula feeding showed a 2.8–4.6-fold higher risk (95% Confidence Interval: Mental Developmental Index 1.73–7.58; Psychomotor Developmental Index 1.44–14.54; body length 1.20–6.41) for developmental deficits (mental and psychomotor developmental index; body length). Developmental care after discharge according to national guidelines did not prevent this. Since this is a retrospective pilot study, no recommendations can be made based on this analysis. Therefore, future research should evaluate whether standard developmental care should be extended by tailored measures depending on individual risk factors.

## 1. Introduction

Over the last two decades, improvements in prenatal, obstetrics, and neonatal care have led to increased survival rates of preterm infants [1,2,3]. One of the most important and remaining challenges is found in the reduction in both neonatal and long-term morbidity [1,2]. The percentage of very premature and very low birth weight preterm infants among all live births in Europe and the USA is 1–2% [4].

Many studies and meta-analyses have investigated either the somatic, psychomotor, or mental outcome of preterm infants, as well as the association with different risk factors. Bronchopulmonary dysplasia (BPD) has been especially shown to have a negative effect on mental development [5]. Additionally, a systematic review and meta-analysis of 15 studies (including 12 randomized controlled trials) with a total of 4984 children and 1416 BPD cases indicated that exclusive feeding with human milk is associated with a significant reduction in BPD risk [6]. Nevertheless, a multi-centric study of the German Neonatal Network (GNN) with 1433 premature infants showed low growth rates for premature infants born at a gestational age under 32 weeks who were exclusively fed with human milk [7]. Yet, another meta-analysis of a total of 16 studies with 1251 preterm infants found limited evidence that preterm formula improves the catch-up growth rate until the age of 18 months [8].

Furthermore, risk factors that may be associated with cognitive impairment and affecting the prognosis of very immature preterm infants include sepsis [9] and intraventricular hemorrhage (IVH) [10]. In addition, Ehrenkranz et al. already observed a lower postnatal weight gain in preterm infants with BPD, necrotizing enterocolitis (NEC), late-onset sepsis, and severe IVH by conducting a large multi-center study with 1600 preterm infants [11]. Klevebro et al. confirmed these results in an international multi-center study with 2521 preterm infants. The study showed significantly worse growth rates within the first 12 postnatal weeks in preterm infants suffering from BPD or NEC [12].

A meta-analysis of 41 articles taking 9653 children into account concluded that prematurity is associated with a significant motor impairment persisting throughout childhood [13]. In addition, preterm infants with poor postnatal catch-up growth seem to be at risk for attention problems throughout school-age [14].

Nevertheless, all these studies investigated either the somatic, psychomotor, or cognitive development of preterm infants. No study showed the combined influence of the risk factors on the long-term somatic, motor, and cognitive outcomes of preterm infants.

The more immature a premature infant is, the more susceptible its brain is to developmental disorders [15]. In contrast to this, high plasticity of organs is assumed in preterm infants, leading to a great ability to change and adapt before a function is finally differentiated [16]. Early developmental care serves to protect the developing brain, including reducing neuronal death after trauma, strengthening, and creating new synapses [17]. Thus, mental and psychomotor outcomes can be improved [14,18,19]. The development of preterm infants can be positively influenced by developmental care [19] and should already be started during the inpatient stay in the neonatal care unit. A meta-analysis of 13 randomized controlled trials showed that developmental care in the neonatal intensive care unit (NICU) could improve both mental and psychomotor developmental outcomes at the age of 12 months in the Bayley Scales of Infant Development II (BSID-II) [17].

However, developmental care varies greatly due to the global and clinical heterogeneity; therefore, more studies are needed to evaluate the effects of developmental care [17]. In the United Kingdom, a guideline on developmental follow-up of preterm infants has been developed similar to the one in Germany. This guideline also includes regular examinations and information on extended developmental care. In this definition, developmental care includes the treatments in the period after discharge [20]. In contrast, the Newborn individualized Developmental Care and Assessment Program (NIDCAP), an individual developmental care program, only takes place in the NICU [21].

Improved selection of high-risk populations must identify those infants who will benefit most from intervention [19]. It remains unclear whether children with different risk factors are at different risks for somatic, psychomotor, or mental developmental deficits.

Thus, the aim of this exploratory study was to investigate the long-term influence of the various risk factors and the benefit of developmental care on the somatic, psychomotor, and mental outcomes of infants born ≤1500 g and/or 32 gestational weeks.

## 2. Materials and Methods

### 2.1. Patients

**Figure 1:**
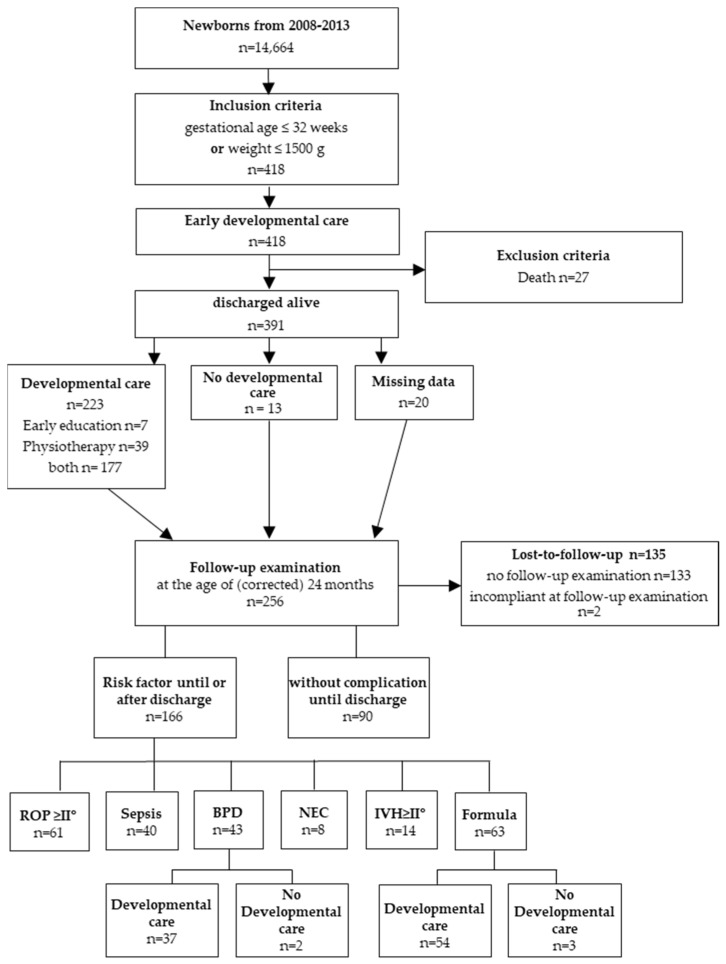
Study design. Overview of the inclusion and exclusion criteria of the study, developmental care, and follow-up examinations. Overall, 418 infants born ≤32 weeks and/or ≤1500 g were identified between 2008 and 2013. Twenty-seven infants met the exclusion criteria. All infants received early developmental care. Follow-up examinations were performed at the age of (corrected) 24 months. Two hundred fifty-six infants were examined, of which 166 showed risk factors until or after discharge from the hospital. Two hundred twenty-three children received developmental care after discharge until the age of 24 months. Abbreviations: *n*: number; BPD: bronchopulmonary dysplasia; IVH: intraventricular hemorrhage; NEC: necrotizing enterocolitis; ROP: retinopathy of prematurity.

The retrospective study was conducted at a tertiary NICU in Rostock, Germany (STROBE-checklist [22], Table S1). The unit accommodates 10 high- and 14 low-care beds. From an annual average of 3000 newborns, about 10% of the children are inpatients. Per year, about 70 of these children are born ≤1500 g and/or 32 gestational weeks. In the period from January 2008 to December 2013, infants born at a gestational age ≤ 32 weeks and/or a gestational weight ≤ 1500 g were identified. Children were included if they matched at least one criterion. Therefore, children > 32 weeks were part of the study if they weighed < 1500 g, or vice versa. Of these, infants discharged alive were included. Data of medical charts were summarized retrospectively regarding baseline characteristics (gender, gestational age, and birth weight) and risk factors (BPD, formula/human milk feeding, NEC, sepsis, retinopathy of prematurity (ROP) ≥ stage 2, and IVH ≥ grade 2). The infants were fed according to the NICU standard operating procedure (SOP) due to the lack of national guidelines for feeding preterm infants. Here, preterm infants < 1500 g were fed with breast milk or donor breast milk, if available. Formula milk if neither breast nor donor breast milk was available. After discharge, counseling for parents on proper nutrition occurred at each follow-up visit. Germany has a list of 32 registered donor breast milk centers, the NICU in Rostock received donor breast milk upon request from three different locations. The dietary classification was performed at 6 months of age. Children never fed with human milk (breast milk or donor breast milk) were categorized as “formula”; children fed with human milk at least once were categorized as “human milk”. There were no children switched from formula milk to human milk. The classification of the mode of feeding was made and reviewed blinded by two independent reviewers (L.N. and J.M.) who were not further involved in the study or the follow-up assessments. Infants who died before discharge have been excluded (Figure 1).

### 2.2. Developmental Care

Since the spectrum of developmental interventions varies worldwide [17], we used current developmental care interventions at our center, which were developed according to national guidelines [23]. Besides common care methods such as diminishing stress and supporting feeding, as well as sleep rhythms, infants born with a gestational age ≤ 32 weeks and/or a gestational weight ≤ 1500 g are referred to daily individual physiotherapy during hospitalization. Each session takes about 20 min and is based on neurophysiological and neurodevelopmental principles. Exercises are aimed inter alia at the prevention of pneumonia, as well as postural anomalies, stimulation of orofacial functions, and promotion of sensorimotor development. Additionally, parents are instructed to perform appropriate exercises, which in turn supports bonding processes between parent and child. Early developmental care is provided to all children in the NICU. Treatments analyzed in this study, also referred to as developmental care or follow-up care were carried out after discharge. Infants continue physiotherapy and are furthermore referred to so-called early intervention, each on a weekly basis. Early intervention is offered to children who have a disability or are at risk of developmental disorders in the first two years of life. It aims to support children and parents to enable individual development of skills and integration into the social environment.

In Germany, so far, uniformly organized developmental care for premature babies after discharge has only been provided to premature babies with a birth weight of less than 1000 g [23]. Based on this, children < 1000 g have a legal entitlement to follow-up examinations. According to the German Federal Social Welfare Code (BSHG), early support is regarded as integration assistance, including curative education measures for children who are not yet of school age with costs covered by health insurance funds. However, national guidelines [23] recommend developmental care for preterm infants < 1500 g. Therefore, it was applied for all of our patients but not always approved by health insurance funds.

### 2.3. Follow-Up Examination

Follow-up examinations were performed at the age of (corrected) 24 months. Baseline characteristics (body weight, height, head circumference) were collected, as well as psychomotor and mental development using the BSID-II according to German guidelines [23]. The BSID, first published in 1969 by Nancy Bayley [24], which was later revised as the BSID-II [25], is the gold standard in assessing neurodevelopment in the first 42 months of life of infants born very preterm and with very low birth weight [4,26]. The BSID-II has been applied by trained physicians. The BSID includes two indices, namely the psychomotor development index (PDI) and mental development index (MDI), composed of 138 and 178 items. In both indices, the mean value in the reference population is 100 (standard deviation (SD) 15); scores above 85 show regular performance, whereas scores of 70–85 show a moderate, and below 70 a severe delay of mental and psychomotor development [25].

Somatic outcomes (body weight, body length, head circumference) were evaluated using national reference percentiles [27]. Sufficient catch-up growth was defined as somatic parameters between the tenth and ninetieth percentiles, whereas moderate growth retardation was defined by somatic parameters between the third and tenth percentiles and severe growth retardation under the third percentile.

### 2.4. Statistics

All statistical analyses were performed using IBM SPSS Statistics for Windows, Versions 25.0 and 27.0 (IBM Corp. Released 2017 and 2020. Armonk, NY, USA). The level of significance was set to 5% (*p* < 0.05). First, the composition of the population was described using descriptive statistics. In a second step, the cohort was then divided and evaluated according to individual risk factors (NEC; ROP ≥ stage 2; BPD; IVH ≥ grade 2, sepsis, and exclusive diet with formula milk). These risk factors were selected based on existing literature and previous results [5,6,9,10,28]. Thirdly, the risk of developmental delay was calculated using a multivariate binary logistic regression, where the cohort was divided into MDI/PDI groups < and ≥ 85 and somatic parameters < and ≥ tenth percentiles. This method calculates the influence of multiple risk factors independently. Finally, the risk factor-specific subgroups were compared regarding the influence of developmental care by applying a multivariate binary logistic regression.

### 2.5. Ethics

The study was approved by the Ethics Committee of the Medical Faculty of the Rostock University, Germany (approval no.: A 2015-0128, A 2015-0178, and A 2020-0207).

## 3. Results

### 3.1. Patients

The characteristics of the study population are shown in Figure 1. From a total of 14,664 births, a series of 418 live births at ≤32 gestational weeks, and/or ≤1500 g were identified. Of these, 391 (94%) were discharged alive (Figure 1). Baseline characteristics of the patients during hospitalization, e.g., gestational age, birth weight, and risk factors, are shown in Table 1. Comparing male and female preterm infants showed no significant differences regarding gender, birth weight, length, and head circumference as well as gestational age.

A total of 256 preterm infants were examined after (corrected) 24 months using the BSID-II (Figure 1). Thus, a lost-to-follow-up of 35% (*n* = 135) must be noted.

193/256 (75%) infants were fed with human milk in their first 6 month of life (at least once), whereas 63/256 children (25%) were fed exclusively with formula milk. ROP ≥ stage 2 occurred in 24% (*n* = 61), sepsis in 16% (*n* = 40), IVH ≥ grade 2 in 5% (*n* = 14), BPD in 17% (*n* = 43) and NEC in 6% (*n* = 15) of the study population (Table 1) The overall mortality rate was 27/418 = 6.5% (Figure 1).

### 3.2. Risk-Factor-Related Outcomes

**Figure 2:**
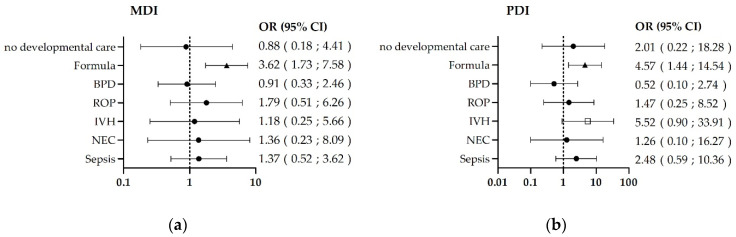

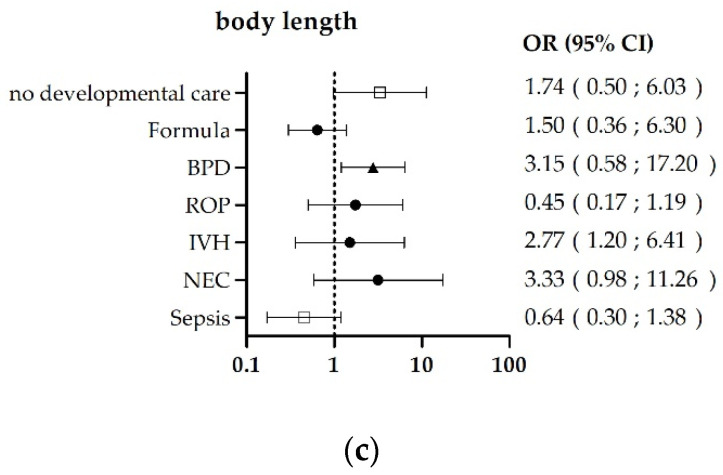
Influence of risk factors on mental, psychomotor, and somatic outcomes of 256/391 children examined after 24 months. Of these, 166 showed risk factors for growth retardation and/or mental and psychomotor deficits. The multivariate regression analysis of the somatic parameters and scores in the Bayles Scales of Infant Development II showed significant deficits (▲). (**a**) Exclusive formula feeding was associated with a 3.62- fold increased risk of developmental deficits in mental (*n* = 43) and (**b**) a 4.57-fold increased risk of developmental deficits in psychomotor development (*n* = 43). (**c**) BPD was associated with a 2.77-fold increased risk of growth retardation (length, *n* = 63). □ *p* > 0.05, ● *p* > 0.12 Abbreviations: MDI: mental developmental index; PDI: psychomotor developmental index; BPD: bronchopulmonary dysplasia; ROP: retinopathy of prematurity; IVH: intraventricular hemorrhage; NEC: necrotizing enterocolitis; OR Odds ratio CI Confidence interval.

An exclusive diet with formula milk was associated with a 3.62 and 4.57-fold risk (95% Confidence Interval (CI) MDI 1.73–7.58; PDI 1.44–14.54, Figure 2a,b and Table S2) of mental and psychomotor developmental delays, respectively (MDI 99 ± 14 vs. 90 ± 15; PDI 102 ± 12, vs. 95 ± 13). BPD was associated with a 2.77-fold (95% CI 1.20–6.41; Figure 2c and Table S2) increased risk of growth retardation (86.4 ± 4.7 vs. 84.1 ± 4.2).

### 3.3. Developmental-Care-Related Outcomes

Overall, 81% (207/256) of the patients in our cohort show an age-appropriate mental and 92% (236/256) an age-appropriate psychomotor development. Nevertheless, a total of 5% (*n* = 12/256) of our cohort showed a severe mental and 3% (*n* = 7/256) severe psychomotor developmental delay. In contrast, every fourth to sixth child showed a severe somatic developmental delay in one or more categories (17–22%, *n* = 43–57) (Table 2). Thirty-nine percent (*n* = 99) showed no somatic, mental, or psychomotor developmental delay, and 65–72% showed age-appropriate growth regarding body weight, length, and head circumference (Table 2).

Only 13/256 infants (5%) did not receive developmental care (Figure 1). All in all, infants without developmental care were not at higher risk for a developmental delay and reached age-appropriate mean values regarding mental (MDI 97 ± 15 and 98 ± 15) and psychomotor (PDI 100 ± 13 and 106 ± 14) development at the age of (corrected) 24 months. Neither infants with BPD nor infants who never received human milk showed differences with and without developmental care (Figure 2 and Table 3). Complete subgroup analyses are shown in the Supplemental Materials (Tables S3 and S4). Because the group of children without developmental care was small, meaningful regression was not possible for some parameters. The complete results of the regression with subgroups can be found in the Supplemental Materials.

## 4. Discussion

In this study, we have presented a unique and comprehensive evaluation of a cohort of 256 children born ≤ 1500 g and/or ≤ 32 gestational weeks over a total of two years regarding the influence of different risk factors on somatic, psychomotor, and mental development.

A total of 9% (*n* = 23/256) of our cohort showed a moderate or severe psychomotor developmental delay. A systematic review of 30 studies with 10,293 preterm infants in 2018 showed an overall psychomotor delay in 21% among all infants [29]. The same systematic review estimated mental developmental delays in 17% of all infants. In our cohort, also 17% of all infants also showed moderate or severe mental deficits. All in all, our observed prevalence of psychomotor and mental developmental delays seems rational against the background of literature.

In our study, ROP ≥ stage 2 occurred in 24%, sepsis in 16%, IVH in 5%, and NEC in 6% of the study population. In contrast to existing literature, these risk factors were not significantly associated with somatic, psychomotor, or mental developmental delays. Eighty-one percent of our cohort showed age-appropriate mental and 92% age-appropriate psychomotor development. However, these risk factors are only partly responsible for the long-term outcome [19]. Income and geographical region were identified as relevant factors of prevalence variability regarding psychomotor and cognitive outcomes of preterm infants [29], as well as parental education, parenting style, parental mental health, family structure, family functioning, and the home environment [19,30]. Moreover, it is known that results in motor [31] and mental development [32] show a wide variability across studies and countries.

In our cohort, BPD and an exclusive diet of formula milk were associated with a significantly higher risk of mental or psychomotor developmental delays. Neither infants with BPD nor infants exclusively fed with formula milk showed differences with and without developmental care. Even though the European Society for Pediatric Gastroenterology, Hepatology and Nutrition Committee on Nutrition recommends breast milk feeding in their position paper [33], there is no guideline for feeding preterm infants. Therefore, the choice of feeding mode was based on the SOP of the NICU in Rostock. Randomization of the feeding mode seemed ethically difficult and was not applicable due to a retrospective study design. Hence, several known and unknown confounders may have had an influence on dietary choices. Despite this, two Cochrane reviews could not find eligible studies comparing breast milk and formula feeding. Rather, the authors pointed out that allocation of nutrition is difficult [34,35]. However, a Cochrane review that analyzed twelve studies comparing donor breast milk and formula concluded that, on the one hand, formula led to an increased size growth during hospitalization, but on the other hand was associated with a higher risk of NEC. No effect on long-term outcomes could be found [36].

Eighty-seven percent (*n* = 223/256) of our cohort attended developmental care, which is consistent with recently published data of follow-up programs across Europe (*n* = 3635). Here, 90% of all children had used (unspecified) “follow-up services” [30]. According to our findings, there is insufficient evidence that the American NIDCAP, in comparison with routine care in preterm and low-birth-weight infants, improves short- and long-term neurodevelopmental outcomes [37]. Despite different studies confirming the benefit of developmental care [17,19,38,39], the diversity of intervention programs leads to significant heterogeneity of outcomes between studies [19]. Particularly the timing in the NICU or after discharge makes a major difference. While some studies only focus on developmental care in the NICU [17], this study only includes developmental care after discharge. In the NICU, all children received the same treatment. The Cochrane review by Spittle et al. also refers to developmental care after discharge [19]. The German guideline on which the follow-up of our cohort is based does not clearly define the type of developmental care. Therefore, no uniform, standardized therapy could be offered to the children after discharge. Thus, the type of therapy was chosen individually for the children depending on the physician and physiotherapist. In conclusion, there is a high need for evidence on optimal developmental care designs while taking individual risk factors into account.

The present study had several limitations. With a lost-to-follow-up of 35%, the outcome could be overestimated in case many of the missing developmental scores were under-age appropriate. Reasons for not attending follow-up visits can be many and varied; according to Little et al., these include problems of mobility and understanding of the disease, as well as incomprehension of the benefits of follow-up [40]. As the sample size of our single-center case series is small and given the retrospective study design, the presented results remain descriptive and should be interpreted with caution. For instance, the group of children without developmental care is small, so some results of the regressions are not meaningful. Further research should follow to confirm these trends. Additionally, the data are only comparable to a limited extent since using the BSID-II leads to significantly higher prevalence rates than the newer version (BSID-III) [29]. In addition, a blind classification was not possible. Even though the classification into dietary groups was carried out very strictly, the possibility of unconscious confounders that influenced the decision still remains. This study provides tentative hypotheses and no conclusive statistics. Since it is a retrospective and not an experimental design, no power analysis has been performed.

This study showed BPD and formula feeding as relevant risk factors for a lower development of preterm infants. As addressed in previous studies, this risk could not be compensated for current developmental care.

## 5. Conclusions

In this study, infants with BPD and an exclusive diet with formula milk were at high risk for developmental delays. Standard developmental care after discharge according to national guidelines could not compensate for these deficits.

Therefore, the question arises whether the standard developmental care should be extended by tailored measures depending on the individual risk factors and needs of the infant and family. Since this is a retrospective pilot study, no recommendations could be made based on this analysis. We anticipated our exploratory study to be a starting point for an evaluation of the national database of the German Neonatal Network (GNN). If the findings are confirmed, an individualized approach should be examined according to the risk factors.

## Figures and Tables

**Table 1:** Study population.

		*n*	%
Female		129	50
**gestational age at birth (weeks)**	mv ± sd	29 ± 2	
<24		3	1
24–27 + 6		77	30
28–31 + 6		157	61
≥32		19	7
**birth weight (g)**	mv ± sd	1187 ± 347	
<750		30	12
750–999		59	23
1000–1499		127	50
≥1500		40	16
head circumference at birth (cm)	mv ± sd	27.0 ± 3.1	
**risk factors**			
exclusively formula until 6 months age		63	26
sepsis until discharge		40	16
BPD until discharge		43	17
IVH until discharge			
grade 1		20	8
grade 2		7	3
grade 3 und 4		7	3
NEC until discharge		8	3
ROP until discharge		61	24
stage 1		44	17
stage 2		11	4
stage 3		6	3

Abbreviations: *n*: number; mv: mean value; sd: standard deviation; BPD: bronchopulmonary dysplasia; IVH: intraventricular hemorrhage; NEC: necrotizing enterocolitis; ROP: retinopathy of prematurity. Baseline characteristics until discharge of all children born ≤32 weeks and/or ≤1500 g between 2008 and 2013, who have been examined at the age of 24 months (*n* = 256). Children, who met at least one criterion were included.

**Table 2:** Developmental deficits and outcome of all infants regarding risk factors at the age of 24 months in BSID-II.

	<85/<10. Perc		≤70/≤3. Perc		Total
	*n*	%	*n*	%	mv ± sd
All (*n*, % = 256, 100%)					
MDI	37	15	7	3	97 ± 15
PDI	12	5	11	4	101 ± 13
Body weight (g)	32	13	57	23	11,354 ± 1693
Body length (cm)	26	10	45	18	86 ± 5
Head circumference (cm)	47	19	43	17	48 ± 2
Formula (*n*, % = 63/27%)					
MDI	23	37	6	10	90 ± 15
PDI	8	13	4	7	95 ± 13
Body weight (g)	22	36	12	19	11,300 ± 1606
Body length (cm)	14	23	6	10	87 ± 4
Head circumference (cm)	26	37	10	16	48 ± 2
Sepsis (*n*, % = 40, 16%)					
MDI	10	25	3	8	95 ± 16
PDI	6	15	3	8	99 ± 17
Body weight (g)	13	33	8	21	10,900 ± 1301
Body length (cm)	10	26	7	18	85 ± 4
Head circumference (cm)	18	46	8	21	47 ± 2
BPD (*n*, % = 43, 17%)					
MDI	10	23	2	5	94 ± 15
PDI	5	12	2	5	98 ± 17
Body weight (g)	21	50	16	38	10,554 ± 1350
Body length (cm)	20	48	14	33	84 ± 4
Head circumference (cm)	24	57	9	21	47 ± 2
IVH ≥ grade 2 (*n*, % = 14, 5%)					
MDI	4	29	2	14	88 ± 19
PDI	3	21	2	14	91 ± 16
Body weight (g)	9	70	6	46	9870 ± 1229
Body length (cm)	6	46	5	38	83 ± 3
Head circumference (cm)	7	54	4	31	47 ± 2
NEC (*n*, % = 8, 3%)					
MDI	3	38	1	13	89 ± 18
PDI	2	25	1	13	92 ± 20
Body weight (g)	4	57	2	29	11,231 ± 1778
Body length (cm)	4	57	3	43	83 ± 4
Head circumference (cm)	1	14	1	14	48 ± 1
ROP ≥ stage 2 (*n*, % = 17, 7%)					
MDI	6	35	2	12	92 ± 18
PDI	3	18	1	6	94 ± 14
Body weight (g)	10	59	5	29	10,435 ± 1504
Body length (cm)	8	47	5	29	83 ± 4
Head circumference (cm)	10	59	6	35	46 ± 2

Abbreviations: BSID-II: Bayley Scales of Infant Development II; Perc: Percentile; *n*: number; mv: mean value; sd: standard deviation; BPD: bronchopulmonary dysplasia; IVH: intraventricular hemorrhage; NEC: necrotizing enterocolitis; ROP: retinopathy of prematurity; MDI: Mental Developmental Index; PDI: Psychomotor Developmental Index.

**Table 3:** Subgroup analysis with BPD children at high risk for developmental deficits, formula at the age of 24 months in BSID-II with and without developmental care at the age of 24 months.

BPD		Developmental Care		OR (95% CI)	*p*-Value
		Without (*n* = 2)	With (*n* = 37)		
MDI	mv ± sd	96 ± 14	94 ± 15	<0.10 *	†
PDI		109 ± 6	98 ± 17	<0.10 *	
Body weight (g)		10,450 ± 2758	10,679 ± 1235	1.21 (0.06; 23.24)	
Body length (cm)		84.3 ± 7.4	84.6 ± 3.9	1.23 (0.06; 23.90)	
Head circumference (cm)		47.3 ± 0.4	46.9 ± 1.4	>100 *	
Formula feeding		Developmental care		OR (95% CI)	*p*-value
		Without (*n* = 3)	With (*n* = 54)		
MDI	mv ± sd	83 ± 10	90 ± 14	1.40 (0.10; 18.86)	†
PDI		94 ± 14	95 ± 13	>100 *	
Body weight (g)		11,250 ± 1465	11,250 ± 1665	0.97 (0.06; 10.37)	
Body length (cm)		85.3 ± 4.3	86.4 ± 4.2	1.85 (0.14; 25.33)	
Head circumference (cm)		47.8 ± 0.7	47.5 ± 1.9	0.53 (0.03; 8.56)	

† Multivariate binary regression analysis at *p* > 0.05; *: CI not applicable. Abbreviations: *n*: number; mv: mean value; sd: standard deviation; OR: odds ratio; CI: Confidence interval; BPD: Bronchopulmonary Dysplasia; MDI: mental developmental index; PDI: psychomotor developmental index.

## Supplementary Materials

The following are available online at https://www.mdpi.com/article/10.3390/children8050394/s1, Table S1: STROBE Statement—Checklist of items that should be included in reports of cohort studies; Table S2: Risk for developmental delay regarding risk factors at the age of 24 month (BSID II); Table S3: Risk for developmental delay in children with BPD with und without developmental delay; Table S4: Risk for developmental delay in children with formula feeding with und without developmental delay.
