# Supplementary material for: Risk Factors of Growth Retardation and Developmental Deficits in Very Preterm Infants in a German Tertiary Neonatal Unit
Source: Children (Basel). 2021 May 14;8(5):394. doi: 10.3390/children8050394 (PMC8156291; doi:10.3390/children8050394)
Supplement: Supplementary file 1 [file children-08-00394-s001.zip › children-1157763-supplementary.pdf]

Supplementary Table S1. STROBE Statement—Checklist of items that should be included in reports of cohort studies.

	Item No	Recommendation	line
**Title and abstract**	1	(*a*) Indicate the study's design with a commonly used term in the title or the abstract	19-20
		(*b*) Provide in the abstract an informative and balanced summary of what was done and what was found	19-28
**Introduction**			
Background/rationale	2	Explain the scientific background and rationale for the investigation being reported	15-19, 35-86
Objectives	3	State specific objectives, including any prespecified hypotheses	87-93
**Methods**			
Study design	4		
Setting	5	Describe the setting, locations, and relevant dates, including periods of recruitment, exposure, follow-up, and data collection	96-106, 146-153
Participants	6	(*a*) Give the eligibility criteria, and the sources and methods of selection of participants. Describe methods of follow-up	96-102
		(*b*) For matched studies, give matching criteria and number of exposed and unexposed	n/a
Variables	7	Clearly define all outcomes, exposures, predictors, potential confounders, and effect modifiers. Give diagnostic criteria, if applicable	113-117 153-162, 140-143
Data sources/ measurement	8*	For each variable of interest, give sources of data and details of methods of assessment (measurement). Describe comparability of assessment methods if there is more than one group	148-163
Bias	9	Describe any efforts to address potential sources of bias	116-118
Study size	10	Explain how the study size was arrived at	98-103
Quantitative variables	11	Explain how quantitative variables were handled in the analyses. If applicable, describe which groupings were chosen and why	156-163
Statistical methods	12	(*a*) Describe all statistical methods, including those used to control for confounding	165-176
		(*b*) Describe any methods used to examine subgroups and interactions	165-176
		(*c*) Explain how missing data were addressed	300-302
		(*d*) If applicable, explain how loss to follow-up was addressed	302-204
		(*e*) Describe any sensitivity analyses	172,176
**Results**			

Participants	13*	(a) Report numbers of individuals at each stage of study—eg numbers potentially eligible, examined for eligibility, confirmed eligible, included in the study, completing follow-up, and analysed	182-189 196-203
		(b) Give reasons for non-participation at each stage	195, 300-302
		(c) Consider use of a flow diagram	195
Descriptive data	14*	(a) Give characteristics of study participants (eg demographic, clinical, social) and information on exposures and potential confounders	205-207, 311-313
		(b) Indicate number of participants with missing data for each variable of interest	195, 231, 300-304
		(c) Summarise follow-up time (eg, average and total amount)	148, 194, 206, 215, 231
Outcome data	15*	Report numbers of outcome events or summary measures over time	230
Main results	16	(*a*) Give unadjusted estimates and, if applicable, confounder-adjusted estimates and their precision (eg, 95% confidence interval). Make clear which confounders were adjusted for and why they were included	213
		(*b*) Report category boundaries when continuous variables were categorized	230
		(*c*) If relevant, consider translating estimates of relative risk into absolute risk for a meaningful time period	n/a
Other analyses	17	Report other analyses done—eg analyses of subgroups and interactions, and sensitivity analyses	233-242
**Discussion**			
Key results	18	Summarise key results with reference to study objectives	248-249 251-253 255-259 266-269 281
Limitations	19	Discuss limitations of the study, taking into account sources of potential bias or imprecision. Discuss both direction and magnitude of any potential bias	298-314
Interpretation	20	Give a cautious overall interpretation of results considering objectives, limitations, multiplicity of analyses, results from similar studies, and other relevant evidence	313-319 320-322
Generalisability	21	Discuss the generalisability (external validity) of the study results	306-308 325-326
**Other information**			
Funding	22	Give the source of funding and the role of the funders for the present study and, if applicable, for the original study on which the present article is based	336

Supplementary Table S2. Risk for developmental delay regarding risk factors at the age of 24 month (BSID II).

**MDI**	**OR**	**95% CI**		**p-value**
ROP ≥ stage II	1.79	0.51	6.26	†
IVH ≥ grade II	1.18	0.25	5.66	
NEC	1.36	0.23	8.09	
Sepsis	1.37	0.52	3.62	
BPD	0.91	0.33	2.46	
No developmental care	0.88	0.18	4.41	
Formula	3.62	1.73	7.58	<0.001
**PDI**	**OR**	**95% CI**		**p-value**
ROP ≥ stage II	1.47	0.25	8.52	†
IVH ≥ grade II	5.52	0.90	33.91	0.07
NEC	1.26	0.10	16.27	†
Sepsis	2.48	0.59	10.36	
BPD	0.52	0.10	2.74	
No developmental care	2.01	0.22	18.28	
Formula	4.57	1.44	14.54	0.01
**Body weight**	**OR**	**95% CI**		**p-value**
ROP ≥ stage II	2.31	0.71	7.58	†
IVH ≥ grade II	2.72	0.67	11.03	
NEC	2.05	0.38	11.05	
Sepsis	0.52	0.21	1.27	
BPD	1.95	0.87	4.37	0.10
No developmental care	2.42	0.73	8.01	†
Formula	0.96	0.48	1.91	
**Body length**	**OR**	**95% CI**		**p-value**
ROP ≥ stage II	1.74	0.50	6.03	†
IVH ≥ grade II	1.50	0.36	6.30	
NEC	3.15	0.58	17.20	
Sepsis	0.45	0.17	1.19	0.11
BPD	2.77	1.20	6.41	0.02
No developmental care	3.33	0.98	11.26	0.05
Formula	0.64	0.30	1.38	†
**Head circumference**	**OR**	**95% CI**		**p-value**
ROP ≥ stage II	1.52	0.47	4.99	†
IVH ≥ grade II	1.47	0.36	5.99	
NEC	0.32	0.03	2.93	
Sepsis	1.36	0.62	2.99	
BPD	1.97	0.90	4.31	0.09
No developmental care	1.18	0.36	3.91	†
Formula	1.05	0.54	2.04	

† p>0.12 (multivariate regression analysis); Abbreviations: BPD bronchopulmonary dysplasia, IVH intraventricular hemorrhage, NEC necrotizing enterocolitis, ROP retinopathy of prematurity, OR Odds ratio, CI Confidence interval, MDI Mental Developmental Index, PDI Psychomotor Developmental Index

Supplementary Table S3. Risk for developmental delay in children with BPD with und without developmental delay.

Children with BPD (n=39)				
**MDI**	**OR**	**95% CI**		**p-value**
ROP ≥ stage II	1.46	0.20	10.60	†
IVH ≥ grade II	0.37	0.03	4.36	
NEC	>100	0.00	*	
Sepsis	2.13	0.35	12.93	
No developmental care	0.00	0.00	*	
Formula	1.98	0.35	11.03	
**PDI**	**OR**	**95% CI**		**p-value**
ROP ≥ stage II	1.53	0.10	23.34	†
IVH ≥ grade II	1.19	0.07	22.07	
NEC	>100	0.00	*	
Sepsis	3.45	0.20	59.10	
No developmental care	0.00	0.00	*	
Formula	0.81	0.05	12.62	
**Body weight**	**OR**	**95% CI**		**p-value**
ROP ≥ stage II	0.66	0.11	4.09	†
IVH ≥ grade II	4.15	0.56	30.59	
NEC	0.66	0.13	3.43	
Sepsis	1.21	0.06	23.25	
No developmental care	0.57	0.13	2.50	
Formula	0.66	0.11	4.09	
**Body length**	**OR**	**95% CI**		**p-value**
ROP ≥ stage II	1.15	0.19	6.78	†
IVH ≥ grade II	1.37	0.21	9.01	
NEC	0.43	0.08	2.29	
Sepsis	1.23	0.06	23.90	
No developmental care	0.50	0.12	2.20	
Formula	1.15	0.19	6.78	
**Head circumference**	**OR**	**95% CI**		**p-value**
ROP ≥ stage II	2.27	0.33	15.77	†
IVH ≥ grade II	6.48	0.59	71.01	
NEC	1.32	0.25	7.07	
Sepsis	>100	0.00	*	
No developmental care	2.45	0.49	12.23	
Formula	2.27	0.33	15.77	

† p>0.05, * CI not applicable; Abbreviations: BPD bronchopulmonary dysplasia, IVH intraventricular hemorrhage, NEC necrotizing enterocolitis, ROP retinopathy of prematurity, OR Odds ratio, CI Confidence interval, MDI Mental Developmental Index, PDI Psychomotor Developmental Index

Supplementary Table S4: Risk for developmental delay in children with formula feeding with und without developmental delay.

Children with formula feeding (n=57)				
	**OR**	**95% CI**		**p-value**
ROP ≥ stage II	2.19	0.25	19.42	†
IVH ≥ grade II	0.31	0.02	6.37	
NEC	1.99	0.09	45.64	
Sepsis	11.54	0.81	164.57	
BPD	0.27	0.03	2.59	
No developmental care	1.40	0.10	18.86	
**PDI**	**OR**	**95% CI**		**p-value**
ROP ≥ stage II	0.00	0.00	*	†
IVH ≥ grade II	0.94	0.03	30.06	
NEC	4.65	0.17	127.43	
Sepsis	>100	0.00	*	
BPD	0.00	0.00	*	
No developmental care	>100	0.00	*	
**Body weight**	**OR**	**95% CI**		**p-value**
ROP ≥ stage II	3.69	0.45	30.20	†
IVH ≥ grade II	0.00	0.00	*	
NEC	>100	0.00	*	
Sepsis	0.33	0.03	3.33	
BPD	2.37	0.43	12.94	
No developmental care	0.79	0.06	10.37	
**Body length**	**OR**	**95% CI**		**p-value**
ROP ≥ stage II	23.80	1.68	337.66	0.02
IVH ≥ grade II	0.00	0.00	*	†
NEC	0.00	0.00	*	
Sepsis	0.36	0.02	5.62	
BPD	2.29	0.35	15.09	
No developmental care	1.85	0.14	25.33	
**Head circumference**	**OR**	**95% CI**		**p-value**
ROP ≥ stage II	2.40	0.28	20.31	†
IVH ≥ grade II	1.83	0.12	27.12	
NEC	0.00	0.00	*	
Sepsis	0.43	0.05	3.93	
BPD	6.42	1.04	39.56	
No developmental care	0.53	0.03	8.56	

† p>0.05, * CI not applicable; Abbreviations: BPD bronchopulmonary dysplasia, IVH intraventricular hemorrhage, NEC necrotizing enterocolitis, ROP retinopathy of prematurity, OR Odds ratio, CI Confidence interval, MDI Mental Developmental Index, PDI Psychomotor Developmental Index
